# Hybrid Printing of Conductive Traces from Bulk Metal for Digital Signals in Intelligent Devices

**DOI:** 10.3390/mi15060750

**Published:** 2024-06-02

**Authors:** Zeba Khan, Addythia Saphala, Sabrina Kartmann, Peter Koltay, Roland Zengerle, Oliver Amft, Zhe Shu

**Affiliations:** 1 Laboratory for MEMS Applications, IMTEK—Department of Microsystems Engineering, University of Freiburg, 79110 Freiburg, Germany; 2 Intelligent Embedded Systems Laboratory, Department of Computer Science, University of Freiburg, 79110 Freiburg, Germany; 3 Hahn-Schickard, 79110 Freiburg, Germany; 4 Actome GmbH, 79110 Freiburg, Germany

**Keywords:** digital signals, molten metal microdroplets, bulk metal, additive manufacturing, wearables

## Abstract

In this article, we explore multi-material additive manufacturing (MMAM) for conductive trace printing using molten metal microdroplets on polymer substrates to enhance digital signal transmission. Investigating microdroplet spread informs design rules for adjacent trace printing. We studied the effects of print distance on trace morphology and resolution, noting that printing distance showed almost no change in the printed trace pitch. Crosstalk interference between adjacent signal traces was analyzed across frequencies and validated both experimentally and through simulation; no crosstalk was visible for printed traces at input frequencies below 600 kHz. Moreover, we demonstrate printed trace reliability against thermal shock, whereby no discontinuation in conductive traces was observed. Our findings establish design guidelines for MMAM electronics, advancing digital signal transmission capabilities.

## 1. Introduction

Additive manufacturing (AM) stands out as a powerful tool, leveraging its rapid and intricate capabilities to efficiently produce smart structures and robust embedded systems [1,2]. Its appeal is particularly strong in fields such as biomedical devices [3], consumer electronics [4], and environmental monitoring [5], where customized sensor electronics tailored to specific objects are crucial. In the realm of electronic boards, the demand for increasingly complex objects [6] and the necessity for ’intelligent’ devices have prompted ongoing research into leveraging 3D printing or additive manufacturing (AM) for their fabrication. While various AM techniques exist for producing conductive and dielectric parts independently, such as direct ink writing (DIW), fused deposition modeling (FDM), material jetting (MJ), stereolithography (SLA), and digital light processing (DLP) [7], the pressing need now is to combine these individual techniques to create MMAM, especially as the requirements for sophisticated electronic functionalities continue to evolve [8]. MMAM facilitates the customization of device design, embedded electronics, and interconnections in a single process, streamlining supply chains and manufacturing [1]. Moreover, MMAM offers solutions for non-geometric objects that are challenging for traditional circuit boards and are often cost-prohibitive, thereby replacing conventional circuit boards and reducing the environmental impact of fabrication methods biased towards chemical waste [9].

Functional electronic boards typically consist of three main components: conductive parts, dielectric parts, and semiconductors. In the development of MMAM, recent years have witnessed a focus on printing conductive parts using nanoparticle-based conductive inks, predominantly deployed through techniques like direct ink write (DIW), inkjet printing, and aerosol jet printing [10,11,12,13,14]. Nanoparticle-based conductive inks typically comprise silver nanoparticles, flakes, or other solid particles dispersed within organic or aqueous solvents [15,16]. Their high conductivity (bulk silver conductivity, $\sigma_{\mathrm{Ag}}=6.3\times 10^{7},S/m$) and thermal stability post-sintering have been attracting attention [17]. However, despite their potential, nanoparticle-based conductive inks often exhibit shortcomings in shape fidelity and electrical performance [2,3,18]. Factors such as nanoparticle size, sintering time, temperature, and ink solution content contribute to these limitations [19]. The sintering temperature for some nanoparticle-based inks is as high as 400 °C [20], which limits the choice of the polymer substrate. Furthermore, nanoparticle-based conductive inks suffer from pattern-dependent resistivity variations due to non-uniform ink drying [21]. When integrated with fused filament fabrication (FFF) for MMAM, these inks can encounter challenges such as interrupted conductance due to disparities in printing resolution and thickness between the FFF-printed polymer and the inks [22]. Improving both the physical and electrical shape fidelity is essential for advancing MMAM’s ability to produce functional electronic boards with heightened performance. Furthermore, there is a need to assess the robustness and reliability of the manufactured parts to ensure their long-term usability.

Our approach differs from traditional ink-based MMAM methods by employing MMAM with molten metal microdroplets and polymers. Demonstrating this technique, we effectively printed low-resistance conductive traces (5.7 mΩ/mm at a trace width of (230 ± 7) μm) on dielectric polymer [23]. These traces exhibited sufficient shear-off adhesion force ((11 ± 5) N), underscoring the successful integration of molten metal microdroplets and polymer dielectric material for MMAM. This paper presents a comprehensive physical and electrical assessment of MMAM-printed parts for their use in functional digital circuit boards.

The conventional design process for digital printed circuit boards involves defining logic, placing components, routing connections, and manufacturing the board [24]. Expanding on this methodology, we introduce physical design guidelines for routing conductive traces on digital MMAM boards. Our investigation includes determining the microdroplet spread factor on polymer substrate, the minimum trace pitch (MTP), and exploring the effect of printing distance on MTP. Additionally, the physical characterization includes assessing the effect of thermal cycling on trace shape fidelity. To evaluate the suitability of metal conductive traces for digital signals, we investigate crosstalk at MTPs across different input signal frequencies using both simulation and experimental models. With the increasing speed of digital systems, understanding the potential presence of signal crosstalk between interconnections in electronic circuits becomes increasingly important [25]. The two main fundamentals of this paper—deposition dynamics of microdroplets on the substrate and signal integrity in digital communication—are explained briefly.

### 1.1. Deposition Dynamics of Microdroplets on Substrate

Molten metal microdroplets are deposited side by side to create metal traces. The deposition behavior of molten metal microdroplets defines the morphology of the then-formed metal trace. The deposition scenario of a microdroplet consists of two stages: the kinematic and the actual deposition [26]. During the kinematic stage, the radius of the drop base remains unaffected by the physical properties of the liquid and the surface. It is only during the actual deposition stage that the influence of these parameters begins to be noticeable [27]. Shin et al. [28], showed that the impact and pile-up microdroplets are influenced by the surface tension (σ), viscosity (η), and density (ρ) of the fluid. The Reynolds number (Re) and the Weber number (We) are utilized to analyze the relative magnitudes of surface tension, viscosity, and inertial forces which are defined as follows [29]:

(1) $$\mathrm{We}=\frac{\rho d_{0}\nu^{2}}{\sigma }$$

(2) Re=ρd0νη

(3) $$\mathrm{Oh}=\frac{\eta }{\sqrt{d_{0}\sigma \rho }}$$

where ν is the impact velocity of the microdroplet, ρ and σ are the density and surface tension parameters of the microdroplet, respectively, η is the dynamic viscosity of the microdroplet, and $d_{0}$ is the initial microdroplet diameter before impact. After being deposited on a substrate, a liquid microdroplet will spread until it reaches a resting equilibrium. At low kinetic energy of the microdroplet, the spreading is governed by the interfacial energy balance as defined by Young’s Equation (4).

(4) $$\sigma_{\mathrm{SV}}=\sigma_{\mathrm{LS}}+\sigma_{\mathrm{LV}}\cos\theta$$

where $\sigma_{\mathrm{SV}}$, $\sigma_{\mathrm{LS}}$, and $\sigma_{\mathrm{LV}}$ represent the substrate-vapor, liquid-substrate, and liquid-vapor surface energies, respectively, while θ denotes the contact angle formed by the microdroplet with the substrate [30]. Assuming volume conservation between the deposited microdroplet and the spherical cap, we can derive the following expression [31]:

(5) $$\beta_{\mathrm{eqm}}={\left(\frac{4}{{\left(1-\cos\theta \right)}^{2}\left(2+\cos\theta \right)}\right)}^{\frac{1}{3}}$$

where $\beta_{\mathrm{eqm}}$ is the dimensionless spread factor of the microdroplet on a substrate, $d_{\mathrm{eqm}}$, normalized to the initial microdroplet diameter, $d_{0}$ and θ is the static contact angle of the microdroplet at the interface of the substrate. On microdroplet solidification, $\beta_{\mathrm{eqm}}$ will define the maximum width of the trace that is formed from the coalescence of microdroplets upon side-by-side deposition. The lateral advance of the metal microdroplets forming the trace and its subsequent contact line is arrested by freezing making the trace grow upward, swelling over the arrested contact line [31]. Furthermore, $\beta_{\mathrm{eqm}}$ will also define the MTP possible on the substrate. Gao et al. [31], described the formation of traces from individual microdroplets. When molten microdroplets are generated at a frequency *f* on a flat surface which is moving at a velocity *U*, the droplets will be deposited with a center-to-center separation:

(6) w=U/f

A line of separate microdroplets will be formed if the *w* is sufficiently large. The microdroplets will overlap if *w* is smaller than the diameter of the base of the sessile droplet when the contact angle is a wetting contact angle. In the event of a non-wetting contact angle, the *w* must be less than the diameter of the curvature of the sessile droplet for individual droplets to form a continuous trace.

### 1.2. Signal Integrity in Digital Communication

Signal integrity refers to the fidelity of an electrical signal. In digital electronics, where data are transmitted and processed in the form of binary values (0 s and 1 s), these values are translated into corresponding voltage or current waveform for transmission and manipulation within the electronic circuits [32]. For short distances and low bit rates, a basic conductor can transmit signals with adequate fidelity. Signal integrity at higher bit rates and over longer distances is chiefly concerned with several key issues, namely reflection, crosstalk, and power or ground noise [33]. Therefore, the signal integrity analysis provides the means to validate design decisions made throughout the design process, including choices regarding components, materials, board layer stack-up, and the creation of net topology. Signal integrity issues typically arise when the system frequency exceeds 50 MHz. Signal integrity engineering fundamentally guarantees the reliable interconnection of electronic circuits, preventing unintended variations in signal quality [34].

Crosstalk refers to the unintended electromagnetic coupling that occurs between traces, wires, trace-to-wire connections, cable assemblies, and components exposed to electromagnetic field disturbances [35,36]. Crosstalk encompasses both capacitive and inductive coupling. Capacitive coupling typically occurs when one trace overlaps another trace, while inductive coupling involves traces situated close to each other [37]. In this scenario, when current flows through one trace (referred to as the aggressor or driven line), it induces a current in the adjacent trace (referred to as the victim line) [38]. The induced current is generated as the signal transmits along the transmission line, and electric and magnetic fields develop between the signaling path and the return path, extending into the surrounding environment [39]. The changing voltage of the aggressor causes noise capacitive coupling current on the jammed line, which is also the victim trace [40]. Given a transmission line length *S* and characteristic impedance $Z_{0}$, with a unit length capacitance between pairs of transmission lines denoted as $C_{\mathrm{ml}}$, an input signal amplitude $V_{\mathrm{input}}$, rise time $T_{r}$, we define the voltage and capacitance between a unit length ΔS as V and $C_{m}$, respectively. Consequently, the induced current is described by the following equation:

(7) $$I=C_{m}\;\cdot \;\frac{\mathrm{dV}}{\mathrm{dt}}=C_{\mathrm{ml}}\;\cdot \;\Delta S\;\cdot \;\frac{V_{\mathrm{input}\;}}{\mathrm{Tr}}$$

Signal jumping is the primary cause of crosstalk. However, in practical design, we can mitigate this negative effect by increasing the spacing between signal lines, employing differential routing techniques, reducing the lengths of parallel runs between signals, and implementing other strategies [34]. In this paper, we only investigate the presence of crosstalk on traces printed adjacent to each other defined by Equation (8).

(8) $$\mathrm{crosstalk}\;\left(\mathrm{dB}\right)=20\log\frac{V_{\mathrm{victim}}}{V_{\mathrm{aggressor}}}$$

where $V_{\mathrm{victim}}$ is the voltage observed at the victim trace, and $V_{\mathrm{aggressor}}$ is the supplied to the aggressor trace.

## 2. Materials and Methods

### 2.1. Hybrid Printing Platform

**Figure 1:**
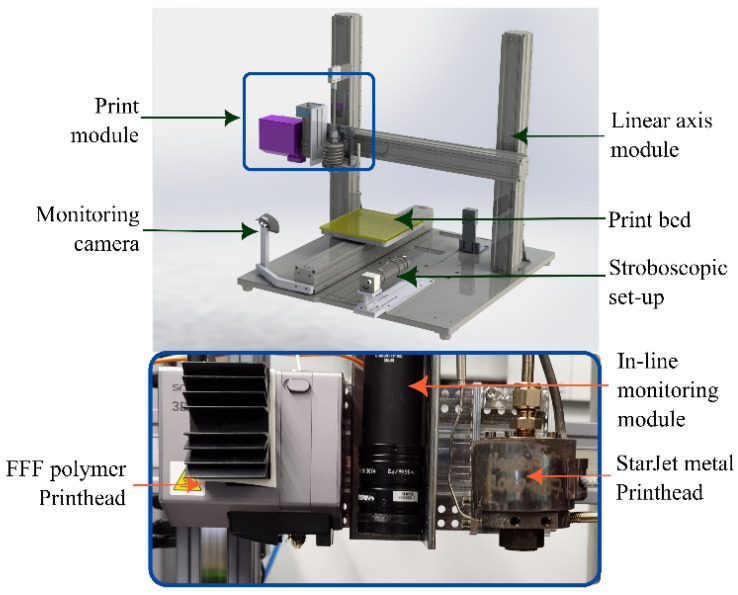
Schematic of our hybrid printing platform showing FFF polymer printhead (top) and StarJet printhead (bottom) mounted on a single axis to enable MMAM printing and the inset shows a photo of the print module containing the two printheads. The printer’s printheads are aligned next to each other along the x-axis, facilitating the sequential printing of metal and polymer materials.

We present the MMAM method for 3D electronics, offering a pioneering approach to hybrid printing of metal and polymer materials. This technique is implemented using an in-house hybrid printing platform [41], as illustrated in Figure 1. The hybrid printer consists of two printheads: one polymer printhead and one metal printhead; see Figure 1 (inset).

The polymer printhead works based on the extrusion principle to deposit molten polymer filament in a layer-by-layer sequential manner on the print bed to form 3D objects (i.e., according to the FFF method). The metal printhead (the StarJet printhead) works on the principle of the pneumatic actuation of molten metal through a small star-shaped orifice. The StarJet printhead is a patented technology of the University of Freiburg, and it is used to print molten metal microdroplets on top of the printed polymer layers to realize the MMAM fabrication method. A comprehensive explanation of the StarJet printhead’s operation can be found in prior studies [42,43]. Sequential printing of the two printheads to generate layer-by-layer hybrid 3D electronics was made possible by hardware, electronic, and software integration explained in detail in our previous work [23]. On our hybrid printing platform, hybrid printed parts necessitate neither pre- nor post-processing, they leave the 3D printer as completely finished electronic boards. Additionally, no chemicals or adhesive promoters are employed. The StarJet printhead utilizes bulk metal in its raw form, presenting an exceptionally cost-effective alternative to state-of-the-art, expensive nanoparticle-based inks [44,45].

### 2.2. Materials and Print Parameters

Polyethylene terephthalate glycol copolymer (PETG) filament (obtained from Prusa Polymers a.s., Prague, Czech Republic) was used to print the polymer substrate. Molten metal microdroplets were printed from flux-free SAC305(Sn96.5Ag3Cu0.5) solder, TAMURA ELSOLD—LEAD-FREE FLUX CORED WIRE, D = 1 mm, Sn96.5Ag3.0Cu0.5, Flux 541 3064BF, 2.2%, 1 KG, (obtained from TAMURA ELSOLD, Ilsenburg, Germany). The polymer filament was stored at room temperature before printing. The filaments did not undergo any active or passive drying processes. There was no heated printing chamber for polymer printing.

### 2.3. Sample Preparation and Fabrication Methodology

A general flow for hybrid sample preparation includes (a) designing polymer substrate and metal traces as computer-aided design (CAD), (b) conversing CAD to Standard Tessellation Language (STL), (c) placing parts in a slicer software in the order of print (layer-by-layer), defining a printhead for each material, (d) defining printing speed for metal printing, (e) and slicing the hybrid model to obtain the G-code. Samples for various investigations were prepared with a fixed substrate thickness of 600 μm, as listed below.

#### 2.3.1. Metal Microdroplets on Polymer Substrates

All the samples were printed at room temperature conditions. A total of 5 samples were generated, and each sample consisted of 10 microdroplets. The microdroplets were printed independently from the raster angle of the polymer trace. The samples were printed by employing the hybrid MMAM technology. The G-code for the hybrid printer consisted of protocols for both polymer and metal printing. First, the polymer substrate was printed, and the metal microdroplets were printed without any delay or subsequent processing steps. In order to ensure that microdroplets did not merge for the analysis, the droplet spacing between microdroplets was kept to 10 times the microdroplet diameter. Additional printing parameters are listed in the Table 1.

#### 2.3.2. Minimum Trace Pitch

We printed a series of five samples featuring adjacent traces, each trace spanning a length of 90 mm. Each sample consisted of five pairs of metal traces. Printing was executed in a continuous sweep, without interruptions for sintering or cooling processes between prints. In this specific scenario, microdroplets needed to be fused to form traces. As a result, the spacing between consecutive microdroplets was set to 100 μm. To print samples with adjacent traces at a fixed pitch, the STL file for metal printing was designed to include traces with a width and thickness of 200 μm, placed side by side at the desired pitch. Each sample comprised only one trace pitch. Metal traces were printed such that each pair of adjacent traces was printed sequentially before moving on to the next pair. This process continued until all five pairs of metal traces were printed. To study how the printing distance of metal microdroplets impacted the pitch of the printed trace, the StarJet printhead was adjusted incrementally in the z-direction for each printing distance variation.

#### 2.3.3. Crosstalk in Adjacent Traces

**Figure 2:**
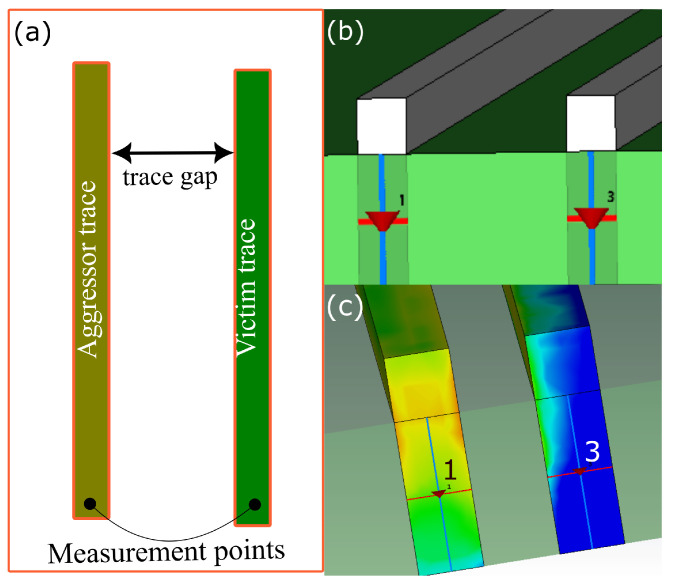
(**a**) Illustration of the crosstalk measurement set for adjacent metal traces, (**b**) simulation model showing the aggressor trace as 1 and the victim trace as 2, (**c**) power flow from the aggressor to victim trace during crosstalk.

Crosstalk analysis was simulated using CST Microwave Studio 2023, with boundary conditions set to open. The analysis employed a design model featuring a PETG substrate measuring 600 μm in thickness. Within this model, metal traces were integrated onto the PETG substrate with a width of 241 μm and an aspect ratio of 1 [23], while maintaining a fixed distance of 281 μm between adjacent traces. The measurements for both the metal trace width and the gap between metal traces were derived from experiments detailed in the results section of this paper. To create a simulation model for crosstalk analysis, one trace was designated as the aggressor trace, and another as the victim trace. For ease of simulation, the ground plane was positioned below the substrate, with energy flow occurring between the trace and the ground plane. This setup was intended to emulate the termination at the ground for both the victim and aggressor traces. When one trace was excited via discrete port 1, the effect could be observed on the inactive port 3, which was located on the adjacent trace (in this case, the victim trace). The voltage observed, denoted as S3,1, denotes the power leaked to port 3 upon exciting port 1, also known as crosstalk; see Figure 2. This is the unintentional signal observed at the victim trace upon exciting the aggressor trace. In the simulation model, the resistivity of the metal trace material was set to 0.3 μΩm while its surface roughness was set to 2 μm. The loss tangent or the tan delta of the polymer substrate was set to 2.1%. The trace was designed with a trace width of 241 μm, a trace height of 280 μm, and a trace length of 90 mm. The thickness of the polymer dielectric substrate was set to 600 μm.

For physical measurements, the PicoScope 7 T&M (Cambridgeshire, UK) was used to read signals from the samples, with a sample rate of 500 mega samples per second. A total of 30 measurements were collected for this analysis. For each input trigger, three waves were considered to read the output voltage on the victim trace. PICO Technology PicoScope 3000 series (Cambridgeshire, UK) hardware was utilized as both a waveform generator and an oscilloscope. The sample was wired such that the aggressor trace received the input voltage, and the readout was connected to the victim trace. Both traces were terminated to the ground.

### 2.4. Data and Image Analysis

The Leica M165C Optical microscope (Leica Microsystems GmbH, Wetzlar, Germany) and the ImageJ software (version 1.54i) were used for imaging and image processing, respectively. A single image was captured from the microscope for each metal trace, and five data points were collected from each image corresponding to trace width data. Each image represented a 1000 μm length of the trace. Given that there were ten metal traces per sample, this resulted in a total of fifty points per sample. Consequently, for each experiment, 250 data points were utilized in total for optical characterization. Metal structures enclosed in polymer underwent cross-sectional analysis by embedding the samples in two-component epoxy to maintain structural integrity. After hardening the epoxy in a pressurized (2 bar) container to remove air gaps, the cross-sectional plane of the samples was exposed using wet grinding. This process employed grit sandpapers of decreasing roughness (180, 320, 600, 1200, 2500, and 4000) with a Struers Knuth Rotor wet-grinder. The procedure included periodic checks with an optical microscope to ensure that the desired cross-sectional plane was achieved. Subsequently, the samples were polished with 9 μm, 6 μm, and 3 μm suspensions (SiC), respectively, followed by image capture using a Zeiss Axioplan microscope with a ProgRes C12 camera (Carl Zeiss AG, Jena, Germany).

### 2.5. Thermal Cycling Test

**Figure 3:**
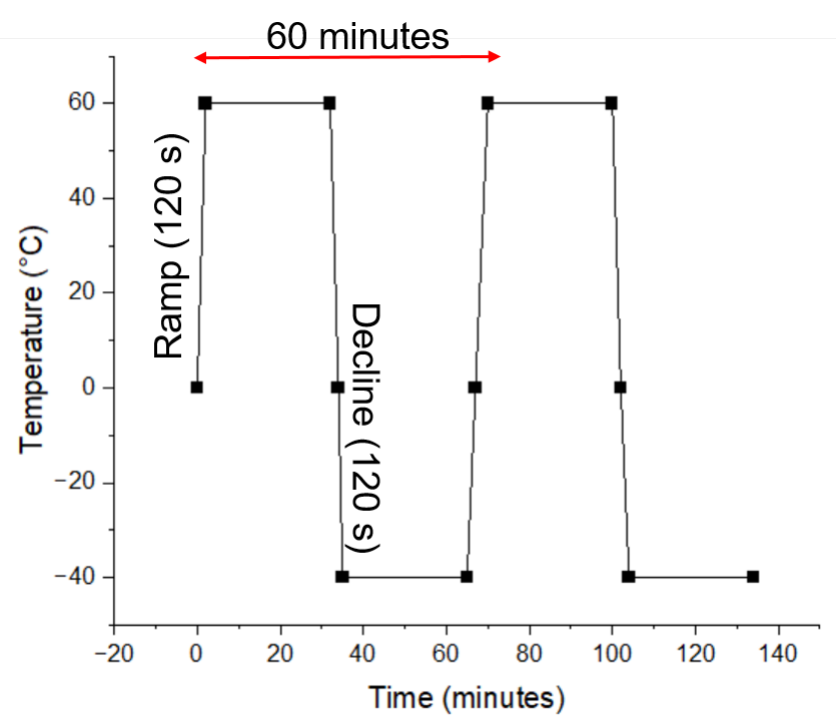
Thermal cycling procedure for hybrid printed samples. The samples underwent temperature cycling between −40 °C and 60 °C with 30-min dwell times at each extreme temperature. The duration of each cycle was 60 min, and a total of 100 cycles was conducted for the analysis of thermal stability.

Thermal cycling of the hybrid printed samples was performed between −40 °C and 60 °C with 30 min of dwell time at the lower extreme and 30 min of dwell time at the higher extreme temperature. The temperature change time was fixed at 120 s during both heating and cooling. Thus, a full thermal cycle took 60 min; see Figure 3. The thermal cycling test was run for 100 cycles (in VOETSCH, Voetsch Industrietechnik, Germany). The higher extreme temperature was limited by the heat deflection temperature of PETG (i.e., 85 °C [46]).

### 2.6. Electrical Resistance Measurement

The four-wire resistance measurement was executed utilizing the KEITHLEY 1100V SourceMeter (Keithley Instruments, Cleveland, Ohio, United States) with KickStart software version 2.11.0 in the current sweep mode. Throughout this process, the current was incrementally increased from 0 A to 1 A, while recording the corresponding voltage drop. One hundred data points were collected per measurement. Subsequently, the resistance of the printed metal structures was calculated based on the resulting Current and voltage curve.

### 2.7. Statistics and Data Analysis

Values represent mean ± standard deviation. The density of the data points is depicted next to the half-box plots. Linear fit with prediction band is represented using 95% prediction interval.

## 3. Results

### 3.1. Quantifying the Spread of Metal Microdroplets on Polymer Substrates

**Figure 4:**
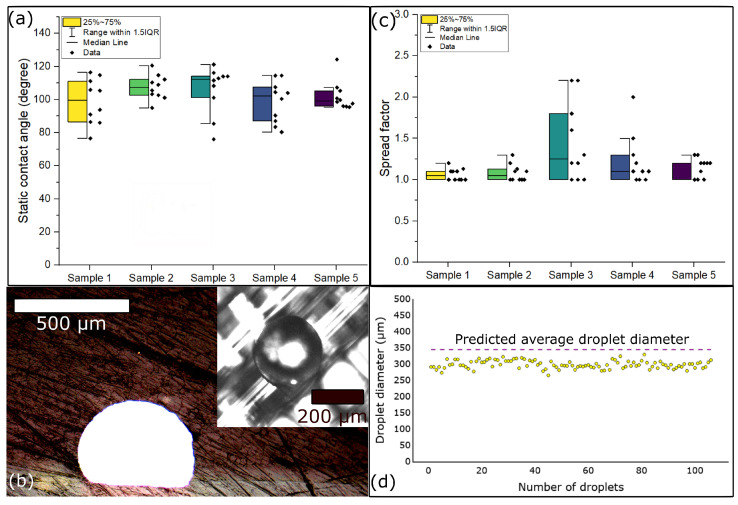
Spread dynamics of metal microdroplets on the polymer. (**a**) The static contact angle of metal microdroplets on the polymer. Each data point is the average of the left and right static angles. The central line within each box represents the median, while the box itself spans the interquartile range, with the upper and lower boundaries of the box marking the 75th and 25th percentiles, respectively. Error bars represent minimum and maximum static contact angles. (**b**) Cross-sectional view of a metal microdroplet deposited with the truncated top on the PETG polymer following the hybrid printing process with an inset view (top right) showing the top view of the metal microdroplet. (**c**) Analytical spread factor of the metal microdroplet on the polymer surface. The central line within each box represents the median, while the box itself spans the interquartile range (IQR), with the upper and lower boundaries of the box marking the 75th and 25th percentiles, respectively. Error bars represent the analytical minimum and maximum spread of the metal microdroplet on the PETG polymer substrate. (**d**) Metal microdroplet diameter measured on the polymer surface (yellow circles) compared with the estimated microdroplet diameter of 341 μm (pink dash line) for 100 metal microdroplets.

With the StarJet printhead, precise control over individual microdroplet generation enables on-demand printing by applying actuation pressure to expel molten metal microdroplets from a star-shaped orifice. Upon impinging on the substrate, the microdroplet can exhibit various behaviors, including splashing, rebounding, or deposition. However, Figure 4b illustrates that the microdroplet predominantly deposits on the substrate without splashing or rebounding, contrasting observations from previous experiments by Rioboo et al. [26]. Rioboo et al. examined the impact of microdroplets on a dry surface, where both the microdroplet and the surface shared the same temperature. In contrast, our study involves metal microdroplets printed from a metal reservoir at an operational temperature of 320 °C onto a polymer substrate situated on a print bed at 85 °C (refer to Table 1). The substantial thermal gradient, combined with the low glass transition temperature ($T_{g}$) of the polymer substrate, may have caused the polymer to melt upon contact with the molten metal microdroplet (the $T_{g}$ of PETG is 85 °C [46]). This phenomenon could have contributed to the rapid solidification at the interface between the metal microdroplet and the polymer substrate, thereby preventing the microdroplet from splashing or rebounding, potentially leading to observations that differ from those reported by Rioboo et al.

Quantifying the spread of deposited microdroplets and predicting their maximum spread post-impact are essential for establishing design rules for printing traces at MTP. Therefore, as described in Section 2.3.1, the contact angles of a total of 100 microdroplets on 5 printed PETG samples were measured. The sessile metal microdroplets (Figure 4b) showcased a diameter of (298 ± 12) μm with a coefficient of variation in a microdroplet diameter of 4%. Figure 4a showcases the measured static solidification angle of the metal microdroplets on the polymer substrate, with a mean static contact angle of (103 ± 4)° and an overall coefficient of variation of 4%. Additionally, the cross-sectional view of the microdroplet (Figure 4b) illustrates its solidified shape, characterized by a truncated top. It is consistent with the neatly deposited microdroplet observed on the polymer surface from the top view.

To further understand microdroplet spreading dynamics, we integrated analytical calculations with experimental data where F. Gao et al. [31] provided insights into the maximum spread of a microdroplet on a substrate under thermal non-equilibrium conditions through Equation (5). By leveraging this equation, we analytically calculated the spread factor of each metal microdroplet on five polymer surfaces, as depicted in Figure 4c.

The analytical calculations revealed a maximum spread of the microdroplet as high as 2.2, with a minimum spread of 1. This implies that the maximum possible diameter of a microdroplet, originally (289 ± 28) μm in air, could extend to 634 μm on the polymer surface. However, due to the non-linear relationship between the static contact angle and the spread factor in Equation (5), the predicted sessile metal microdroplet diameter is the average of the diameter from all individual spread factors obtained in Figure 4c times the microdroplet diameter in air ((289 ± 28) μm), as illustrated by the dashed line in Figure 4d.

The spreading of printed metal microdroplets in our hybrid printing method not only provides valuable insights into predicting the minimum pitch but also serves as a crucial foundation for optimizing the printing process for MTP. With a maximum sessile metal microdroplet diameter of 329 μm, our experimental data forms the basis for estimating the minimum pitch, which we anticipate could extend to approximately 341 μm which is the predicted average sessile metal microdroplet diameter shown as a dashed line in Figure 4d. It is important to note that none of the solidified sessile metal microdroplets observed on the polymer substrate (which are the yellow circles in Figure 4d) reached the average predicted sessile metal microdroplet diameter shown by the dashed line. So, it should be safe to assume that the dashed line represents an upper limit for the possible spreading or size of sessile metal microdroplets on the considered substrate.

### 3.2. Characterization and Optimization of MTP Parameters in Hybrid Printing Methods

In defining the MTP, we aimed to ascertain the minimum pitch, where no printed traces contact each other, achieving 100% efficacy. This parameter holds significant importance in establishing design rules for printing in densely populated electrical circuits. For this investigation, the printing distance (i.e., the distance between the nozzle of the StarJet printhead and the polymer substrate) is kept at 4 mm.

**Figure 5:**
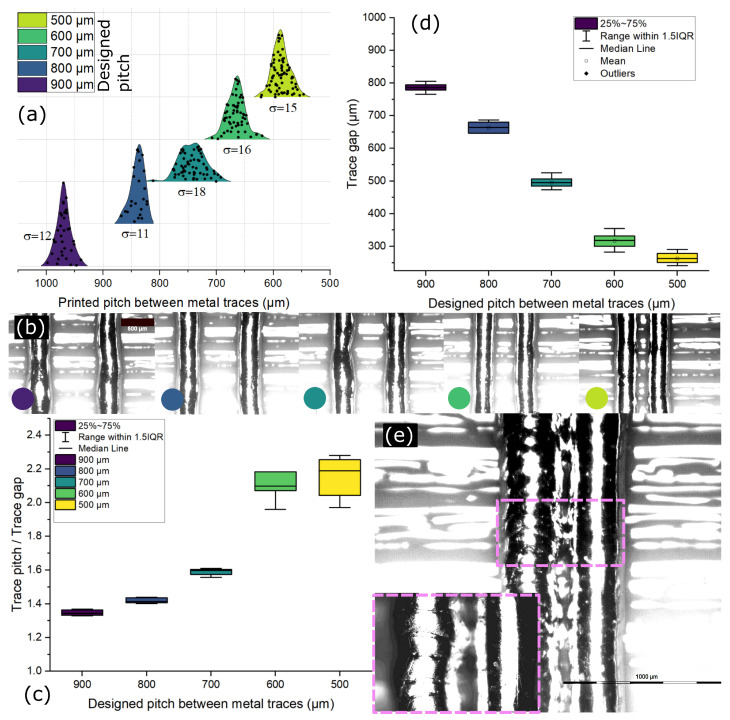
(**a**) Printed pitch distribution curve for the designed pitch versus the printed trace pitch, (**b**) microscopic images of the traces printed at different designed pitches. The color on the image relates to the distribution curve from (**a**), (**c**) the designed pitch of the traces against the ratio of trace pitch to trace gap, (**d**) trace gap of the printed traces obtained for different designed pitches, (**e**) Microscopic image of two traces printed at a designed pitch of 450 μm, which is lower than the MTP, showing a short-circuit between them. The inset provides a magnified view of the section highlighted in the image (**e**), showing the difference in topography of the traces when printed below the MTP, inset shows the magnified image of the traces touching each other as well as the difference in the morphology of the two traces.

**Figure A1:**
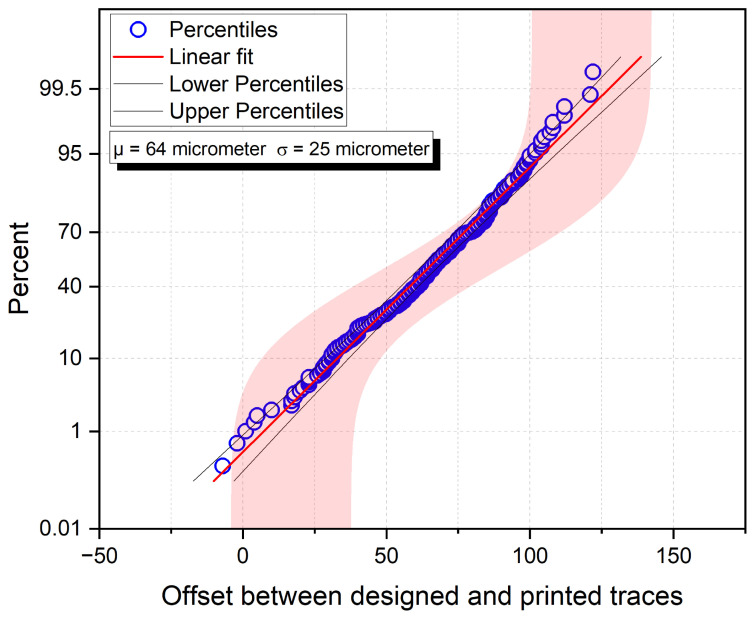
Prediction curve for trace pitch printability.

In this investigation, we pursued a methodical approach to ascertain both the minimum traceable pitch (MTP) and the offset between the printed and designed MTP. Our experimental design involved incrementally varying the pitch between traces, starting from 1000 μm, until the point where traces began to ‘short’ with each other. Leveraging insights from previous results regarding microdroplet diameter on polymer substrates, we anticipated the MTP to converge around the value of 341 μm. Figure 5a illustrates the outcomes of our MTP experiment across the x-axis, ranging from 1000 μm to 500 μm, which represents the designed pitch. Beyond this range, at a designed value of 400 μm, shorting between parallel traces became apparent, as depicted in Figure 5e. Consequently, we limited our results display to pitches of up to 500 μm. Notably, the experimentally determined MTP surpassed the predicted value of 341 μm. To clarify this discrepancy, we plotted a prediction curve for the MTP experiment results against their corresponding designed values, revealing an offset of (64 ± 25) μm (see Appendix A, Figure A1). Despite this offset, a substantial difference of 245 μm persisted between the predicted and experimentally achieved MTP, measured at (586 ± 15) μm.

To delve deeper into the underlying causes of this difference, we analyzed the designed trace pitch against the ratio of trace pitch to trace gap. Remarkably, as the designed pitch narrowed, the deviation in the ratio increased significantly. While this variation was not evident in the trace pitch standard deviation (Figure 5a), it suggested potential fluctuations in the trace gap. Figure 5d further illustrates how the deviation in the trace gap amplifies as the trace pitch decreases. Moreover, at a designed pitch of 400 μm, where shorting between traces occurred, we observed a distinct morphology of the traces, differing notably from those depicted in Figure 5b. Figure 5e provides visual insights into the printed parallel traces at a design pitch of 450 μm, showcasing shorts between traces and a morphology distinct from that observed in Figure 5b. What is particularly noteworthy is the clear boundary of the microdroplets forming the trace, hinting at rapid solidification occurring within the traces themselves rather than solely at the polymer substrate interface when printed at a design pitch of 450 μm. At MTP of (586 ± 15) μm, the minimum trace gap obtained was (281 ± 15) μm. We observed only a 5% standard deviation in the trace gap, which affirms the trace shape fidelity and reliability in trace dimensions for further electrical characterization.

### 3.3. Effect of Printing Distance on Trace Pitch and Trace Gap

In this section, we investigate the effect of printing distance on trace pitch and trace gap. For this experiment, the designed pitch is 700 μm. There is a 200 μm buffer compared to the design pitch of MTP (i.e., 500 µm). Therefore, we can check if the shorting of lines occurs at higher printing distances. The printing distance is defined as the absolute distance between the StarJet nozzle and the top surface of the printed substrate during non-contact trace printing. In this investigation, we increased the printing distance from the standard 4 mm to 12 mm with a step size of 2 mm.

**Figure 6:**
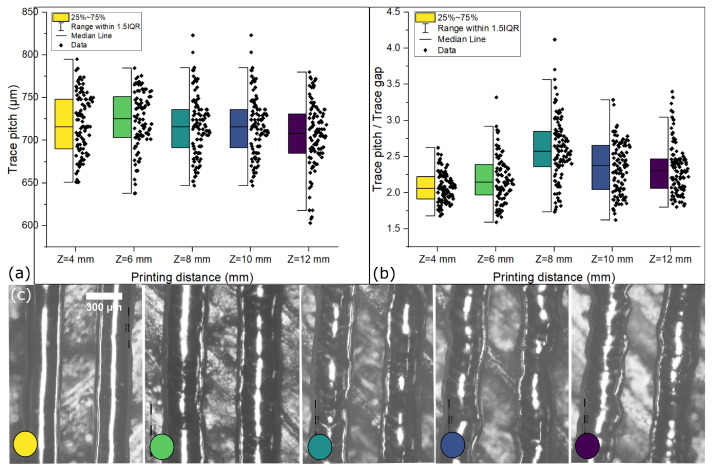
(**a**) Trace pitch printed at different printing distances, (**b**) ratio of trace pitch to trace gap for traces printed at different printing distances highlighting the variation induced from variations in trace gap. (**c**) Microscopic images of traces printed at a design pitch of 700 μm at various printing distances. The color circles on each image correspond to the box colors in (**a**,**b**).

Figure 6a demonstrates minimal variation in trace pitch and standard deviation across different printing distances, suggesting that printing distance has little effect on trace pitch. This outcome showcases remarkable microdroplet deposition precision, even at considerable distances such as 12 mm, a feat challenging to achieve in drop-on-demand printing technologies [47]. However, while consistency in trace pitch is an essential criterion, it does not provide a complete picture of the printing distance’s influence on trace characteristics.

In Figure 6b, a different aspect of the relationship emerges: the standard deviation in the trace gap increases with the increasing printing distance, from 37 μm at 4 mm to 55 μm at 12 mm. Plotting the trace pitch over the trace gap against the printing distance accentuates this trend, highlighting the widening gap deviation as the printing distance increases (see Figure 6c), a phenomenon distinct from the consistent trace pitch observed in Figure 6a.

Furthermore, at a printing distance of 8 mm, the ratio of trace pitch to trace gap peaks, and is likely due to uneven solidification and spreading induced by higher impact velocity. Higher impact velocity implies a higher cooling rate by increased heat dissipation and, therefore, increased solidification. However, beyond this threshold, the ratio diminishes, suggesting that microdroplets solidify before subsequent ones are deposited, resulting in reduced spreading and, consequently, reduced gap deviation. Nonetheless, this also implies that traces formed beyond the 8 mm printing distance may exhibit heightened irregularities in morphology due to reduced microdroplet spreading. Indeed, the analysis indicates that a printing distance of 4 mm exhibits minimal deviation in both trace gap and trace pitch, suggesting its suitability for achieving consistent trace morphology. Although the printing distance does not significantly affect trace pitch, the observed deviations in the trace gap underscore its influence on trace uniformity. Contrary to expectations, increasing the printing distance does not lead to a reduction in the minimum trace pitch obtained at 4 mm. This implies that despite maintaining trace pitch consistency, higher printing distances do not contribute to reducing the minimum trace pitch previously achieved. Therefore, while printing distance plays a crucial role in trace uniformity, it does not necessarily facilitate the optimization of the minimum trace pitch beyond the level achieved at 4 mm.

### 3.4. Crosstalk Characterization between Printed Electrical Traces at MTP

In this section, we characterize the electrical parameters of the traces printed at MTP. For digital signal integrity analysis, we examine the crosstalk present in traces printed adjacent to each other at the MTP.

The investigation into crosstalk involved both simulation and experimental analyses across a frequency range spanning from 100 kHz to 1000 kHz. In the simulation setup, the ground plane was positioned beneath the substrate, facilitating energy flow between the traces and the ground plane. This configuration allowed for the observation of crosstalk, where the excitation of one trace (referred to as the aggressor trace) influenced the adjacent trace (the victim trace), resulting in power leakage to the victim trace.

**Figure 7:**
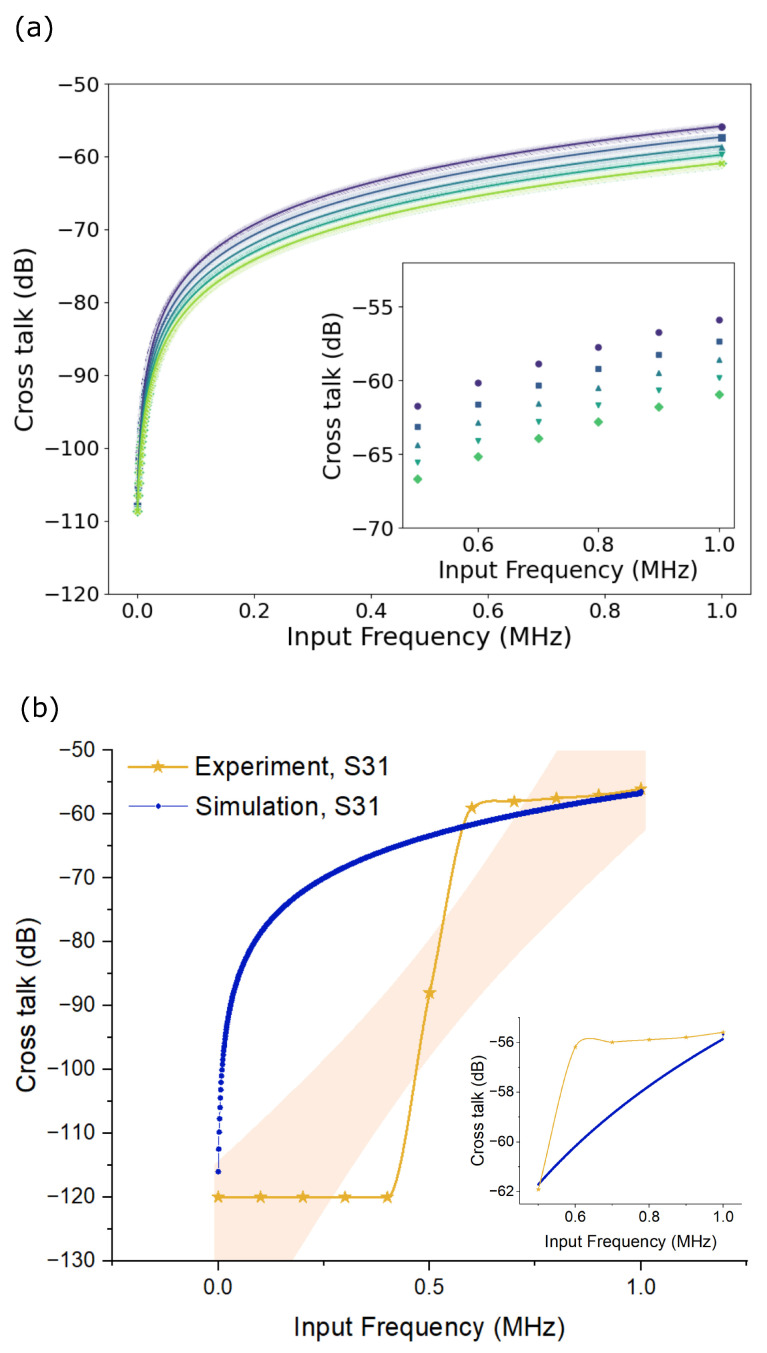
(**a**) Simulation results of trace crosstalk shown for varying trace gaps. The purple line represents the trace gap of the printed sample, 281 μm, with an increment of 150 μm in subsequent lines to show the effect of the trace gap on crosstalk, (**b**) simulated crosstalk versus measured crosstalk on printed traces.

Simulated crosstalk results, depicted in Figure 7a, showcased variations across five different trace gap models. Notably, as the trace gap decreased from a maximum of 781 μm to a minimum of 281 μm, the observed crosstalk between traces increased. At the smallest trace gap of (281 ± 15) μm, the simulated crosstalk at 1000 kHz reached −55 dB. Subsequently, experimental investigations focused on the minimum trace gap obtained during manufacturing trace patterns (MTPs), which was 281 μm. The experimental crosstalk results, as illustrated in Figure 7b, revealed a presence of crosstalk reaching −120 dB up to 600 kHz. This crosstalk, predominantly perceived as noise due to the absence of discernible waveform signals at the victim trace voltage, transitioned into clear waveform signals in the range of −60 dB to −55 dB beyond 600 kHz. Consequently, the findings suggest that traces with widths of (241 ± 12) μm and lengths of 90 mm on a 600 μm-thick PETG dielectric substrate exhibited low resistance ((285 ± 6) mΩ) and experienced limited crosstalk effects, with crosstalk attenuated to −120 dB up to a working frequency of 600 kHz.

### 3.5. Thermal Characterization of the Printed Traces

**Figure 8:**
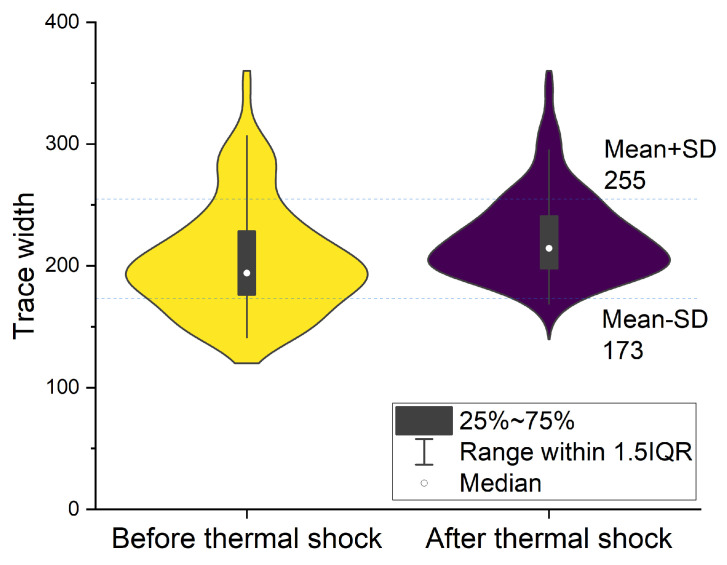
Influence of thermal shock on the printed trace width.

Thermal cycling of the hybrid printed samples was performed between −40 °C and 60 °C with 30 min of dwell time at the lower extreme and 30 min of dwell time at the higher temperature extreme. Thus, a full thermal cycle took 60 min. The thermal cycling test was run for 100 cycles. The effect of thermal shock on printed traces with the above-mentioned defined printing parameters was investigated to assess its impact on trace width and resistance. Upon subjecting the printed traces to thermal shock, we observed a notable change in the mean trace width, increasing from (241 ± 12) μm to (251 ± 16) μm, representing a 4.1% variation, see Figure 8. This change in trace width suggests potential modifications in the polymer substrate and/or the metal traces induced by thermal stress. Remarkably, despite the thermal shock, no detachment of individual traces from the polymer surface was observed, indicating robust adhesion between the metal and polymer components. This favorable adhesion quality suggests promising applications for hybrid printing in the realm of printed electronics, particularly in environments with operating temperatures below 60 °C, leveraging the selected PETG polymer. Furthermore, the ability to modify printing materials by transitioning to polymers with higher glass transition temperatures offers versatility, facilitating the production of both undisturbed electrical traces and polymer components in integrated systems. Such findings underscore the adaptability and potential market applications of hybrid printing technologies in diverse electronic and engineering contexts.

### 3.6. MMAM Printing of Customized Smart Eyeglasses Using Metal Microdroplets and Polymer

**Figure 9:**
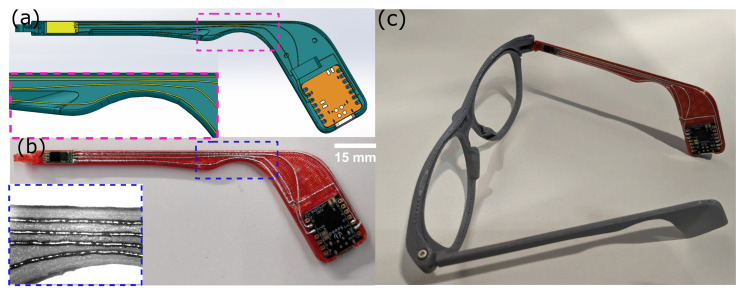
(**a**) CAD model of the smart eyeglass arm, highlighting the point of interest where traces must be printed according to MTP results (inset shows the magnified section of the arm of eyeglass where customized traces are designed which take the shape of the frame), (**b**) printed model of the smart eyeglass arm with traces printed according to MTP at the highlighted point of interest region (see inset), (**c**) a full smart eyeglasses model with electronics interconnected to the printed metal traces.

**Figure A2:**
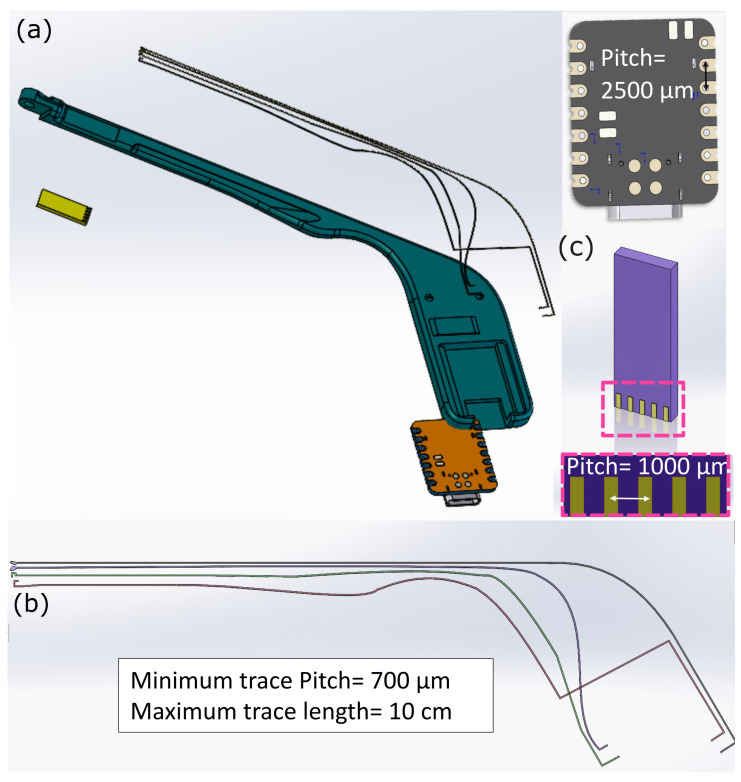
(**a**) CAD model of the smart eyeglass arm in a zoomed-in view showing the 4 interconnecting traces, customized proximity sensor (yellow), microcontroller (orange), (**b**) interconnecting trace dimensions for the designed model (inset ), and (**c**) the pitch between the pins of the microcontroller and proximity sensor (inset highlights the pitch between the pins).

In this section, we showcase a practical application of our MMAM printing technology, leveraging insights gleaned from the MTP analysis presented earlier. Our demonstration entails the creation of a customized smart eyeglass [48], integrating advanced functionalities for sensing applications. One of the eyeglass arms is fabricated using our MMAM printing technology, housing a customized proximity sensor and a microcontroller board interconnected by printed metal traces, as depicted in Figure 9a. Notably, the traces are strategically positioned to conform to the unique shape and topology of the eyeglass arm, as highlighted in the inset of the figure. The designed MTP for this model was 700 μm, a value well within the safe range determined by our MTP analysis. Figure 9b illustrates the printed eyeglass arm model with the integrated traces, faithfully replicating the design from Figure 9a. The workflow and printing process of a hybrid model on our platform has been described in detail in our previous work [41]. The CAD model of the eyeglass arm and the electronics placement can be found in Appendix B Figure A2. The inset image in Figure 9b underscores the precision of the printing process, with traces maintaining a consistent distance from each other despite the varying topography of the eyeglass arm. Our demonstration highlights the synergistic capabilities of additive manufacturing and our MMAM technology, enabling the precise fabrication of metal traces according to customized designs. We have also demonstrated digital communication capabilities by implementing a printed interconnection between the proximity sensor and the microcontroller board on the smart eyeglasses, as shown in Figure 8c). Importantly, the smart eyeglasses deliver comparable functional performance to traditionally fabricated smart glasses, further demonstrating the versatility and potential of the hybrid manufacturing approach [49].

## 4. Discussion

We presented the hybrid printing of conductive traces using molten metal microdroplets from bulk metal to create digital signal tracks in intelligent devices. Our investigation covered the deposition dynamics of metal microdroplets on polymer surfaces, the printing of MTP conductive traces, and the effect of printing distance on these traces. We also studied signal integrity in printed conductive traces through crosstalk analysis using both simulations and experiments. We performed a thermal shock analysis to test the robustness of our hybrid printed electronics and showcased a customized smart eyeglass model to demonstrate real-life applications.

The study of deposition dynamics revealed that the experimentally measured microdroplet diameter was consistently below the analytical value, confirming the reliability of our findings. This alignment ensures accuracy in determining the minimum pitch between traces, optimizing the MTP printing process. Our investigation highlighted the complexities in determining MTP in hybrid printing. While we anticipated an MTP of around 341 μm based on microdroplet diameter and contact angles, our experiment yielded a larger value of (586 ± 15) μm on PETG polymer. This discrepancy underscores the multifaceted nature of printing dynamics, where other factors must be considered to explain variations in trace pitch and gap. The study of printing distance effects showed that increased distance did not change the trace pitch but increased gap variability due to the higher impact velocity of the droplets, aligning with findings by Mohan et al. Regarding signal integrity, the comparison between experimental and simulated crosstalk results showed close alignment around 1 MHz, confirming the simulation model’s suitability for predicting crosstalk in this range. These traces are promising for real-case testing in the (100 to 600) kHz frequency range.

We aim to enhance the versatility of electrical and mechanical designs for 3D electronics, including printing on surfaces like hemispheres, soft polymers, flexible polymers, and irregular 3D objects. This capability could enable the fabrication of complex shapes with integrated electronics, pushing the boundaries of 3D printing for electronics.

## 5. Conclusions

Our MMAM printing technology offers a distinct alternative to the current state-of-the-art nanoparticle-based conductive inks for printing 3D electronics. Unlike conventional methods, MMAM enables the printing of 3D electronics without the need for additional sintering processes or surface treatments on polymer substrates. In this paper, we delve into the potential of MMAM for facilitating digital signal transmission through metal and polymer composites.

We investigated how metal microdroplets spread on polymer surfaces, revealing that an upper limit for the actual spread of printed microdroplets was consistently predictable by the model proposed by Gao et al. [31]. This alignment between experimental and analytical model calculations provides valuable insights into defining the microdroplet spread on various substrates, thereby enabling substrate-specific design rule formation. Furthermore, we established design rules for printing traces at the minimum pitch, with experimental validation revealing an offset being required between the designed pitch and the actual pitch. Interestingly, the experimentally obtained minimum trace pitch differed considerably from that predicted by microdroplet spread experiments, suggesting that the printing of metal traces near is influenced not only by substrate parameters but also by previously printed traces and potentially other factors. Further investigation is warranted to understand the role of previously printed traces in the printing of new traces near, the role of the microdroplet velocity, and the effective cooling process of the traces. Additionally, we explored the effect of printing distance on adjacent traces and found that trace pitch remained unaffected, highlighting the precision and positioning of metal microdroplets by the StarJet technology. However, deviations in trace gaps beyond a certain printing distance could be determined experimentally, as well. They might be attributed to uneven solidification and the spreading of microdroplets with increased impact velocity. Future research will investigate the effect of changing frequency on printing distance and frequency to enable higher printing distances for specific applications.

Moreover, our crosstalk examination in adjacent printed traces advanced our understanding of digital signal transmission in printed electronics. The measured data are closely aligned with simulated data, indicating high fidelity in printed samples and experiments. Furthermore, our results demonstrated a wide frequency range up to 600 kHz for a trace gap of (281 ± 15) μm for exploring digital signal transmission without noise, paving the way for smart 3D electronics such as smart sensors and wearables. As exemplified by our demonstration of a smart eyeglass, future work will focus on real-time analysis of smart 3D electronics to highlight their potential for customized, environmentally friendly, flexible, and application-oriented approaches compared to traditional electronics.

## Figures and Tables

**Table 1:** Various printing and design parameters.

StarJet Parameters	Value	FFF Parameters	Value
Reservoir temperature	320 °C	Polymer road width	450 μm
Orifice diameter	183 μm	Nozzle diameter	400 μm
Printing frequency	18 HZ	Substrate thickness	600 μm
Droplet spacing	2800 μm	Extruder nozzle temperature	240 °C
Printing distance	4 mm	Print bed temperature	85 °C

## Data Availability

The data that support the findings of this study are available from the corresponding author upon reasonable request.

## Abbreviations

The following abbreviations are used in this manuscript:

MMAM	multi-material additive manufacturing
MTP	minimum trace pitch
PETG	polyethylene terephthalate glycol copolymer
SAC305	Sn96.5Ag3Cu0.5
FFF	Fused filament fabrication
CAD	computer-aided design

## Appendix A. Prediction Curve for Offset in Printing MTP

We predict the dimensional accuracy of hybrid printing by comparing the printed trace pitch with the designed trace pitch. When applying a linear fit to the designed vs. printed pitch data, we obtain a prediction of (64 ± 25) µm as the offset in the dimensional accuracy of the printing system; see Figure A1. Using the prediction curve, we can define the design parameter for the trace pitch. This will benefit the design of electrical traces and the definition of semiconductor pin pitch for integrating 3D electronics on our printing platform.

## Appendix B. Model for Smart Eyeglass Arm

The CAD model of the Smart eyeglass arm was created using SOLIDWORKS software (version 16000). The designed width was 200 μm and the aspect ratio of trace width to trace height was designed to be 1. The CAD model for the proximity sensor was designed to assess the position of the traces interconnecting the proximity sensor to the microcontroller; see Figure A2.
